# Sexual Function in Men Living With a Fontan Circulation

**DOI:** 10.3389/fped.2021.765380

**Published:** 2021-11-17

**Authors:** Imants Rubenis, Derek Tran, Andrew Bullock, Vishva Wijesekera, David Baker, Yves d'Udekem, Karin du Plessis, Darren Katz, Michael Lowy, Dominica Zentner, David Celermajer, Rachael Cordina

**Affiliations:** ^1^Concord Repatriation General Hospital, Concord, NSW, Australia; ^2^School of Medicine, The University of Sydney, Camperdown, NSW, Australia; ^3^Heart Research Institute, Newtown, NSW, Australia; ^4^Perth Children's Hospital, Nedlands, WA, Australia; ^5^Prince Charles Hospital, Chermside, QLD, Australia; ^6^Royal Prince Alfred Hospital, Camperdown, NSW, Australia; ^7^Children's National Hospital, Washington, DC, United States; ^8^Murdoch Children's Research Institute, Melbourne, VIC, Australia; ^9^The Royal Children's Hospital, Melbourne, VIC, Australia; ^10^Men's Health Clinic Melbourne, Melbourne, VIC, Australia; ^11^School of Medicine, The University of Melbourne, Parkville, VIC, Australia; ^12^Men's Health Clinic, Double Bay, NSW, Australia; ^13^Royal Melbourne Hospital, Parkville, VIC, Australia

**Keywords:** congenital heart disease, Fontan, sexual health, fertility, erectile dysfunction

## Abstract

**Introduction:** It is unknown if the Fontan circulation has an impact on sexual health in men. This study assessed self-reported sexual health and fertility in men with a Fontan circulation.

**Aims:** In this prospective, cross-sectional study, Australian men ≥18 years enrolled in the Fontan Registry of Australia and New Zealand were invited to complete the International Index of Erectile Function (IIEF), alongside questions assessing fertility. These data were compared to historical, age-matched controls.

**Results:** Of 227 eligible men, 54 completed the survey; of those 37 were sexually active and included in the final analysis. Mean age was 28 ± 3 years, age at Fontan was 5 ± 3 years. Fontan type was extra-cardiac conduit in 15 (41%), lateral tunnel in 12 (32%), and atriopulmonary connection (APC) in 10 (27%). Ventricular function was normal in 24 (83%), and all were New York Heart Association Class I (23 patients, 79%) and II (six patients, 21%). Nine participants (24%) had erectile dysfunction (IIEF-EF score ≤25). The severity was mild (IIEF 22–24) in six (16%), mild–moderate (IIEF 17–21) in two (5%), and moderate (IIEF 11–16) in one (3%). Baseline characteristics and current medication usage were similar in those with and without erectile dysfunction. Compared with historical control values, erectile function was not significantly impaired in the Fontan population (*p* =0.76). Men with a Fontan circulation had decreased levels of sexual desire and overall satisfaction (*p* < 0.001). There was no correlation between the presence of erectile dysfunction and any assessed parameter. Eleven (30%) of the cohort reported a pregnancy with a prior partner.

**Conclusion:** In our cohort, overall erectile function was comparable between men with a Fontan circulation and historical controls, however sexual desire and overall satisfaction were reduced. There was no correlation between study parameters and the presence of erectile dysfunction. The proportion of the cohort who had a prior pregnancy was congruent with population data.

## Introduction

Since its initial development almost 50 years ago, the Fontan procedure has been increasingly implemented for children who have complex single ventricle cardiac anatomy (1–4). Over this time the technique has undergone multiple advancements, including the lateral tunnel (LT) and extracardiac conduit (EC) modifications (2–4). These new techniques, along with improved critical and medical care, have contributed to improved long-term survival for people living with a Fontan circulation—the majority of whom now reach adulthood (5–7).

There are numerous long-term morbidities which are well documented complications of a Fontan circulation due to the altered physiological state characterized by systemic venous hypertension, reduced cardiac output, endothelial dysfunction, neurohormonal activation and chronic cyanosis in many (8). Late complications include increased propensity to thromboembolic events, arrhythmia, and cyanosis as well as Fontan-associated liver disease, protein losing enteropathy and plastic bronchitis (5, 8). The impact that a Fontan circulation has upon sexual function, satisfaction and fertility is less well established. A proportion of women with a Fontan can conceive and carry a pregnancy safely through to delivery but fertility is reduced (9–11). The effect that Fontan physiology has upon sexual function in either sex is not well characterized and fertility within the male population is not established. We sought to investigate male sexual function in young adult men from the Australia and New Zealand Fontan Registry.

## Methods

For this prospective, cross-sectional study, men over the age of 18 years who had consented to involvement in research were contacted *via* the Fontan Registry of Australia and New Zealand. A total of 227 patients across all Australian states were identified and contacted between March 2019 and July 2020. A mailed questionnaire was sent to eligible men. If no response was received, they were contacted via telephone and given the opportunity to complete the questionnaire online. The study was approved by the Human Research Ethics Committee at all involved institutions (HREC2019.042).

The survey utilized the International Index of Erectile Function (IIEF), an internationally validated sexual health questionnaire. The IIEF examines the domains of erectile function, orgasmic function, sexual desire, intercourse satisfaction and overall satisfaction. Questions were also included to provide a qualitative assessment of fertility. Patients self-reported appropriate scores for each domain based on their experience in the preceding 4 weeks. The IIEF defines a diagnosis of erectile dysfunction (ED) as an erectile function score of ≤26 (out of a possible 30). Mild ED is defined as an IIEF-EF score of 22–25, mild-moderate 17–21, moderate 12–16, and severe <11. These data were compared with historical control data with mean age of 35 years (12).

Men were excluded if they had a history of significant mental health disorder, or if they were not sexually active prior to the study period. Baseline characteristics were obtained from records of the Fontan Registry of Australia and New Zealand.

Erectile function score and the presence of erectile dysfunction were analyzed according to patient age, age at Fontan, years since Fontan, type of type of Fontan, NYHA class, oxygen saturations and ventricular function, and prior pregnancy in a partner. Clinical variables were used if collected within 12 months of questionnaire completion. Continuous variables were compared utilizing Student's independent or one sample T test and reported as mean ± standard deviation. Proportions were analyzed using a Chi-square test and reported as number (%). Correlations were completed using Spearman rank correlation and Pearson correlation coefficient. All statistical analysis was completed via SPSS v26 for Windows. Statistical significance was based on a *p* value of < 0.05.

## Results

Of 227 eligible men, 135 were unable to be contacted or did not complete the survey, 22 were excluded due to a history of mental disorder, 17 refused to complete the survey upon contact, 13 did not have sexual activity during the study period, two declined to have their registry data accessed, and one person died during the study period, leaving 37 men included in the final analysis.

Baseline characteristics are shown in Table 1 and were evenly matched between those without and with erectile dysfunction. Four men in total had implantable cardiac devices, all of which were in the group without ED (*p* = 0.23). Fourteen patients in total had a history of arrhythmia, eight in the cohort without ED and six in the cohort with ED (*p* = 0.75). All arrhythmias were atrial in origin.

**Table 1:** Baseline characteristics.

	***n***	**No ED**	***n***	**ED**	***p***
Age, years (±SD)	28	29.4 ± 6.2	9	27.4 ± 4.3	0.39
Fontan type, *n* (%) (APC vs. TCPC)					0.62
AP		7 (25%)		3 (33.3%)	
LT		9 (32.1%)		3 (33.3%)	
ECC		12 (42.1%)		3 (33.3%)	
Age at Fontan, years (±SD)	28	5.2 ± 3.0	9	5.6 ± 2.8	0.67
Years since Fontan (±SD)	28	24.2 ± 6.6	9	21.9 ± 4.6	0.34
Ventricular function, *n* (%)^a^	23		6		0.24
Normal/mild		20 (87%)		4 (66.7%)	
Moderate/severe		3 (13%)		2 (33.3%)	
NYHA class, *n* (%)^a^	24		5		0.97
Class I		19 (79.2%)		4 (80%)	
Class II		5 (20.8%)		1 (20%)	
Implantable cardiac device, *n* (%)	28	4 (14.3%)	9	0 (0%)	0.23
Resting oxygen saturations, % (±SD)^b^	20	92.4 ± 5.0	6	93.0 ± 3.3	0.72
History of arrhythmia, *n* (%)		8		6	0.75
Medications, *n* (%)					
Warfarin		16 (57.1%)		3 (33.3%)	0.21
Aspirin		10 (35.7%)		2 (22.2%)	0.45
ACEI		10 (35.7%)		2 (22.2%)	0.45
DOAC		0		2 (22.2%)	0.01
Spironolactone		2 (7.1%)		0	0.41
Sildenafil		1 (3.6%)		0	0.57
Digoxin		2 (7.1%)		1 (11.1%)	0.70
Sotalol		4 (14.3%)		2 (22.2%)	0.57

*Continuous data analyzed utilizing Student's Independent T Test. Continuous variables reported as mean ± standard deviation, categorical data reported as number and percentage (%) and analyzed using Chi-square test*.

a *Data unavailable for 8 patients*.

b *Data unavailable for 11 patients*.

*AP, atriopulmonary connection; LT, lateral tunnel; ECC, extracardiac conduit; NYHA, New York Heart Association Functional Class; ACEI, angiotensin converting enzyme inhibitor; DOAC, direct oral anticoagulant*.

Patients with a Fontan circulation had significantly decreased levels of sexual desire at 8.1 ± 1.2 compared to 9.1 ± 1.0 (*p* < 0.001) and overall satisfaction at 8.3 ± 1.9 compared to 9.5 ± 0.8 in controls (both out of 10) (*p* < 0.001).

**Figure 1:**
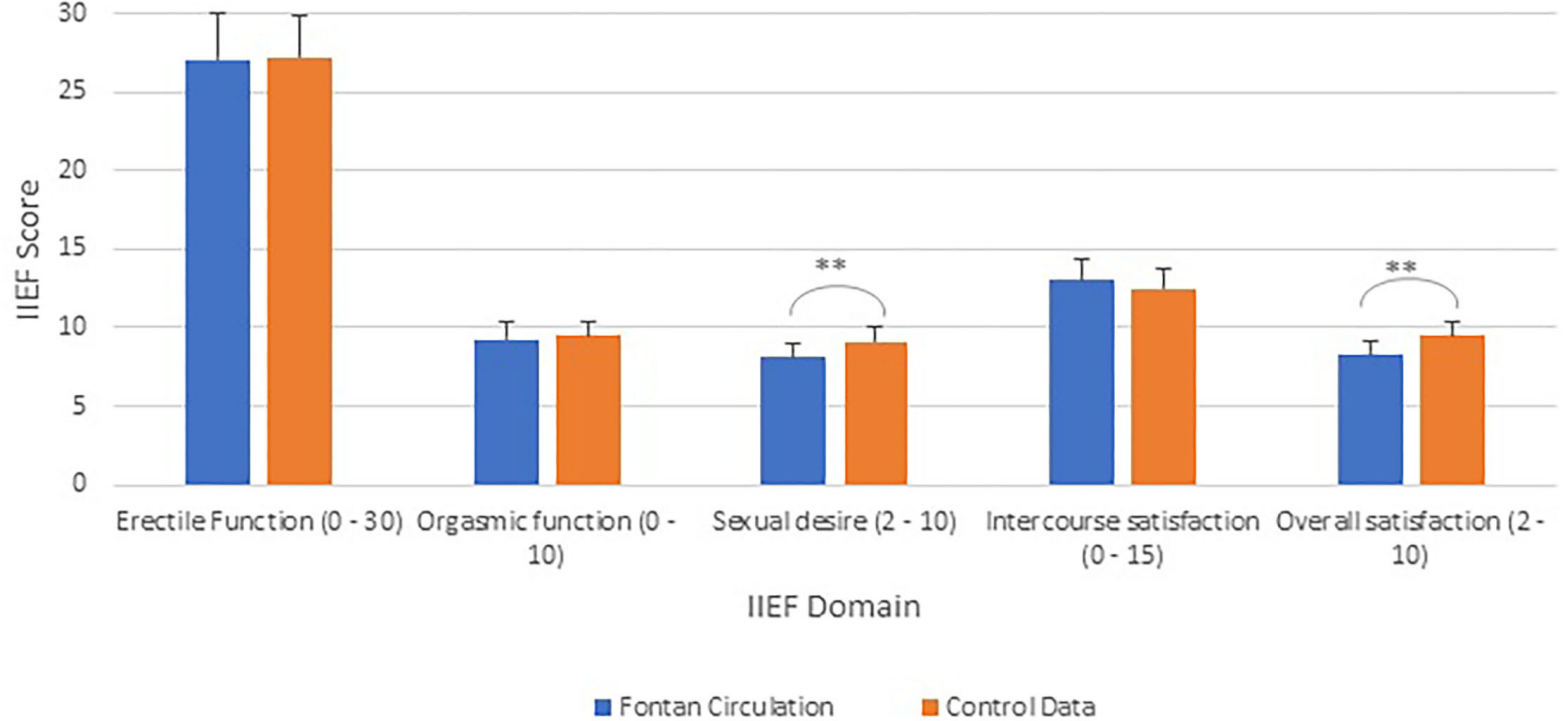
International Index of Erectile Function scores (IIEF) (range) for men with a Fontan compared to reference control data (12). **p < 0.001.

As demonstrated in Figure 1, the average IIEF erectile function score was 27.1 ± 3.9 (out of 30) for the Fontan cohort, compared to 27.2 ± 3.2 for historical control data (*p* = 0.76). Table 2 outlines the results for the IIEF domains for those with ED compared to those without ED. As expected, those with ED had significantly reduced levels of erectile function (*p* < 0.001). Patients with ED also had significantly decreased scores in the domains of orgasmic function (*p* = 0.05), intercourse satisfaction (*p* = 0.01), and overall satisfaction (*p* = 0.01). There was no difference between groups in sexual desire (*p* = 0.29).

**Table 2:** IIEF results comparing those with ED to those without.

**IIEF Domain (range) (m ± SD)**	**No ED (*n* = 28)**	**ED (*n* = 9)**	***p***
Erectile function (0–30)	28.8 ± 1.4	21.4 ± 4.3	<0.001
Orgasmic function (2–10)	9.8 ± 0.6	7.8 ± 2.6	0.05
Sexual desire (0–10)	8.2 ± 1.3	7.6 ± 1.1	0.29
Intercourse satisfaction (0–15)	13.6 ± 1.7	11.1 ± 2.4	0.01
Overall satisfaction (2–10)	8.6 ± 1.5	7.1 ± 1.2	0.01

*Continuous data analyzed using Student's independent sample T test. All variables reported as mean ± standard deviation*.

*ED, erectile dysfunction*.

**Figure 2:**
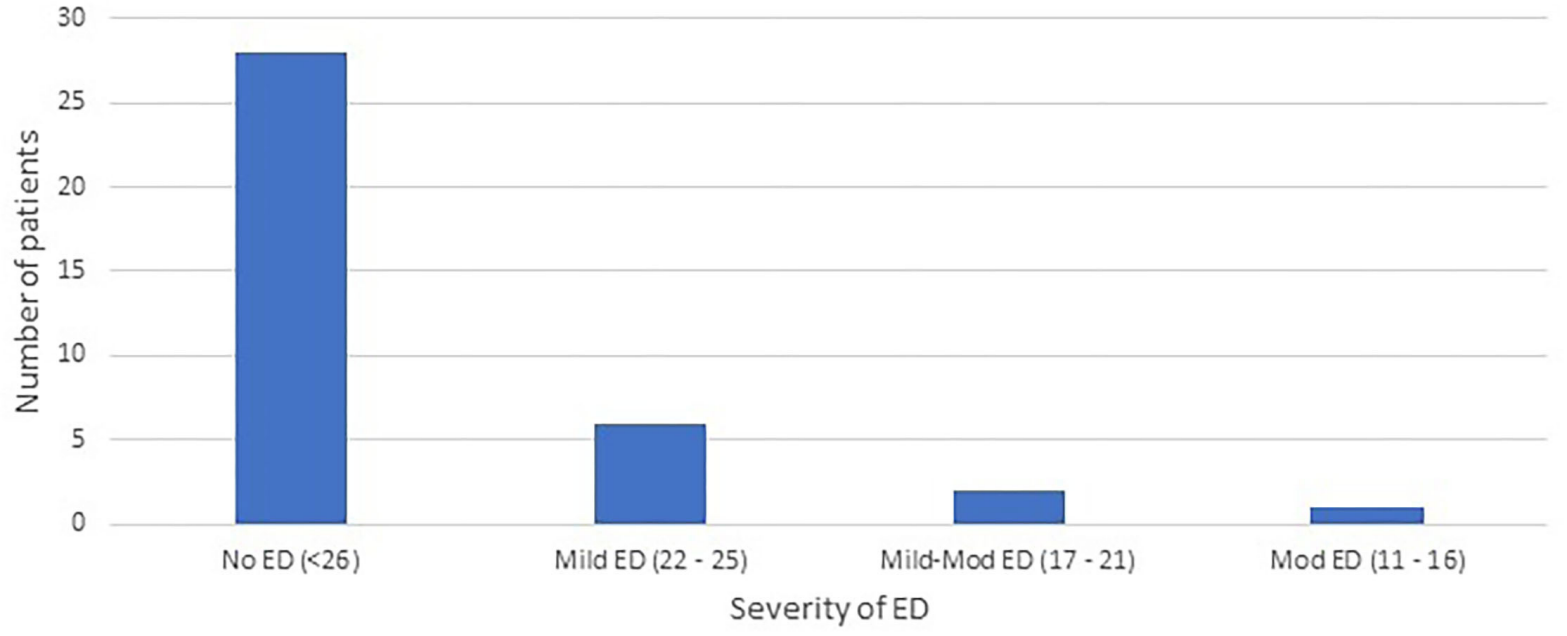
Number of patients with, and severity of erectile dysfunction (ED) *via* the International Index of Erectile Function.

The range of IIEF-EF scores are demonstrated in Figure 2. Nine participants in the study (24%) reported an IIEF-EF score consistent with a diagnosis of erectile dysfunction. Of these, severity was mild (IIEF-EF 22–25) in six (16%), mild-moderate (IIEF-EF 17–21) in two (5%), and moderate (IIEF-EF 11–16) in one (3%).

There was no difference in orgasmic function (*p* = 0.44) and intercourse satisfaction (*p* = 0.14). There was no association between clinical indices and IIEF-EF (Table 3; *p* > 0.1 for all). There was no difference in IIEF-EF score between those with APC compared to those with total cavopulmonary connection (TCPC; *p* = 0.65).

**Table 3:** Correlation of IIEF Erectile Function Score and presence of ED against type of Fontan, NYHA FC, ventricular function, and prior pregnancy.

**Variable**	***n***	***r***	***p***
Age, years	37	0.16	0.34
Age at Fontan, years	37	0.01	0.96
Years since procedure	37	0.15	0.38
Type of Fontan procedure (APC vs. TCPC)	37	−0.08	0.64
NYHA FC	29	−0.01	0.97
Ventricular function	29	0.22	0.26
Resting oxygen saturations	26	−0.11	0.60
Prior pregnancy	11	0.70	0.68

*Non-parametric data analyzed using presence of ED via Spearman rank correlation. Continuous data analyzed using Pearson correlation coefficient with EF score*.

*TCPC, total cardiopulmonary connection*.

A total of 11 men (30%) in the cohort reported a pregnancy in a prior sexual partner. There was no significant difference in the proportion of those with and without ED who reported a prior pregnancy (*p* = 0.85). Of those who did not report a pregnancy, 19 (51%) reported they had only ever used contraception. Four men (11%) reported they had been trying to conceive with a partner for greater than 6 months. There was no difference in IIEF-EF score between the patients who had and those who had not reported a prior pregnancy (*p* = 0.41). Patients who had an APC Fontan procedure were more likely to report a pregnancy compared to those who underwent a TCPC procedure (*p* < 0.001).

## Discussion

As Fontan-specific management has refined, an ever-greater proportion of the population are surviving well into adulthood. Consequently, important issues are arising that were not contemplated in an earlier era focused on early life (13, 14). Holistic care should include guidance for adults regarding the impact that their unique physiology may have on sexual function, and the ability to have children.

In this cross-sectional study we present the largest prospective report of the subjective sexual health and self-reported fertility of men with a Fontan circulation to date. We utilized the IIEF tool. We found that almost one quarter of men studied within our cohort had IIEF-EF scores consistent with a diagnosis of erectile dysfunction. We compared the IIEF-EF scores of our cohort to those of historical age-matched controls. Even though 24% of our population had IIEF-EF scores indicating ED, overall, the EF score for the cohort not significantly different to that of the control data. Our data demonstrated that most of the cohort had well preserved erectile function, and that those with ED did not suffer from a severe phenotype. This is congruent with reports more broadly in congenital heart disease (CHD) literature that ED is typically of mild severity (15). Prior studies which have utilized the IIEF in the setting of (CHD) reported rates of ED to be between 10–14% (15, 16). However, when including studies that have included different assessment tools, rates of ED ranged from 10% up to 38% (17, 18), with reported risk varying from the same as expected “normal” population rates to two times higher (19, 20). In Australia, ED rates in young men have been reported to be lower than the rates we recorded in young men living with a Fontan circulation; Chew et al. (21) found that in a cohort of men aged 20–29 years the background rate of ED in the community was 16 and 8.7% in 30–39 year olds using the IIEF-5 questionnaire (a truncated version of the IIEF utilized in this study, with similar sensitivity and specificity).

Most studies that have examined sexuality in the setting of CHD have included heterogenous CHD types without distinction as to the underlying pathology or anatomy; this is an important consideration for the Fontan population who are affected by unique physiological problems including chronic venous hypertension and reduced cardiac output, especially during exertion. Elevated systemic vascular resistance, chronic cyanosis and cardiovascular drugs including anti-arrhythmics and some diuretics (spironolactone in particular) may also contribute to dysfunction (18, 22).

Wolff et al. (10) are the only group who have examined sexual wellbeing in a small group of men (*n* = 7) with a Fontan circulation. This study included a questionnaire and interview assessing sexual satisfaction and fertility (10). The issues reported included avoidance of sexual activity, that may in part be related to symptoms during exertion (17, 23). Consistent with the results of our study, there was no significant difference in the presence of erectile dysfunction when patients with a Fontan were compared to controls (10). Two out of seven (29%) of patients in their cohort reported erectile dysfunction, a result congruent to the 24% in our cohort (10).

Completion of the IIEF allowed assessment of sexual health across multiple domains. Our results demonstrated that when compared with healthy controls, men with a Fontan circulation had decreased levels of sexual desire and overall satisfaction. The domains of sexual health, such as desire and satisfaction, are less well studied than erectile function, though have been identified to potentially be reduced in patients with cardiac disease (16, 24). Deficiencies in these areas are likely multifactorial in nature. Patients with CHD have reported the fear of developing cardiac symptoms during sexual intercourse as detrimental to overall sexual health (15, 25). Many CHD patients develop health concerns from a young age. It has been theorized that the presence of significant illness during adolescence may be detrimental to sexual development, and indeed may lead to avoidance of sexual activity (15, 23). Our results are congruent with a prior study which demonstrated men with CHD had a decreased level of overall satisfaction and orgasmic function (16). However, this study of pooled, predominantly simple CHD did not demonstrate any difference in level of sexual desire. In contrast, other research did not find a significant difference in sexual satisfaction in a heterogenous group of men with CHD compared to healthy controls (23)—clearly, more well-designed data are needed. Of the prior CHD studies in this field, men with a Fontan circulation were infrequently included (15, 17, 26).

Long-term medical therapy is frequently required for the management of a person with a Fontan circulation. The effect that this has on sexual health of men has not been clearly delineated. Men with CHD have previously been found to attribute ED to prescribed medical therapy (17). Spironolactone is often cited as being contributory due to its known androgen-suppressing properties, and it has been theorized that prescription of spironolactone may lead to impaired sexual functioning for men with CHD (10, 15, 18). In a cohort of adult male patients aged in their 40s with mixed CHD, the use of spironolactone and digoxin were associated with sexual dysfunction (18). In our cohort spironolactone was only prescribed in two men (5%) and so, although we did not note an association with erectile dysfunction our data in that regard are limited. Beyond the field of CHD, the exact impact that spironolactone has on the sexual health of men with acquired cardiac disease has not been conclusively defined (27). Beta-blockers and ACE inhibitors are classes of medication which have previously been associated with sexual dysfunction in men with CHD in some (17, 23) but not all studies, in keeping with our findings (16, 18).

Our study demonstrated that approximately 30% of surveyed men reported a pregnancy in a partner and there was no association between IIEF-EF score and prior pregnancy. We did note that patients with an APC were more likely to have reported a prior pregnancy, a finding which must be interpreted with caution given the limited numbers involved. Four patients reported unsuccessful conception with a partner for longer than 6 months. Census data have demonstrated that between 17 and 35% of the population at a comparative age have fathered children (28). A single prior study examined fertility in the setting of a Fontan circulation (10). Of seven surveyed men in this study, two had previously fathered children (10). Based on limited data, there is currently no evidence that the Fontan circulation has an impact on the male ability to father children.

There are limitations to our study. Of the 227 eligible patients, 135 (60%) were either unable to be contacted or did not complete the survey. A number of these had outdated contact details. Others never returned the survey despite repeated contact. Thus, selection bias is an important consideration. It is feasible that due to the personal nature of the questioning, men with erectile dysfunction may have been less inclined to complete the survey. Men with more significant erectile dysfunction may also have a propensity to avoid sexual intercourse, and thus have been excluded from final analysis once the survey was completed. The overall small number of patients within both cohorts makes it important to consider that the study was underpowered to detect true differences in baseline characteristics between those with and without erectile dysfunction. It is theorized however, that chronic venous hypertension may be implicated in the pathogenesis of sexual dysfunction in men with a Fontan circulation. As our study did not incorporate invasive haemodynamic assessment, we have been unable to assess whether this is contributory.

A further limitation to research in sexual health is the reliance on self-reported questionnaires. It is also noted that several different research tools have been used in this area. We utilized the IIEF, as it has a broad base of supporting literature and has been used previously in the field of adult congenital heart disease (15, 16). Other studies have been performed utilizing other tools for assessment of erectile dysfunction or sexual dysfunction. These include the Golombock-Rust Instrument of Sexual Satisfaction, or the IIEF-5. The diversity in assessment tools utilized is a hinderance to researchers attempting to draw direct comparisons between results of different studies.

## Conclusion

In our cohort, erectile function was comparable in men with a Fontan circulation compared with age-matched historical controls, though they did report decreased levels of sexual desire and overall satisfaction. Overall, men with a Fontan circulation reported levels of fertility congruent with that of the general population. Clinicians should be aware that erectile dysfunction may occur in this population and consider asking appropriate questions to ensure that concerns regarding sexual health and fertility are not overlooked.

## Data Availability Statement

The raw data supporting the conclusions of this article will be made available by the authors, without undue reservation.

## Ethics Statement

The studies involving human participants were reviewed and approved by HREC Royal Children's Hospital, Parkville, Victoria. The patients/participants provided their written informed consent to participate in this study.

## Author Contributions

RC: concept design, project supervision, and data analysis. IR: assistance with concept design, survey implementation, and data analysis. DT, AB, VW, DB, Yd'U, KP, DK, ML, DZ, and DC: assistance with project design, data interpretation, and manuscript preparation. All authors contributed to the article and approved the submitted version.

## Conflict of Interest

The authors declare that the research was conducted in the absence of any commercial or financial relationships that could be construed as a potential conflict of interest.

## Publisher's Note

All claims expressed in this article are solely those of the authors and do not necessarily represent those of their affiliated organizations, or those of the publisher, the editors and the reviewers. Any product that may be evaluated in this article, or claim that may be made by its manufacturer, is not guaranteed or endorsed by the publisher.

## Abbreviations

ED: erectile dysfunction
EF: erectile function
IIEF: International Index of Erectile Function
NYHA: New York Heart Association
CHD: congenital heart disease
APC: atriopulmonary connection
LT: lateral tunnel
ECC: extracardiac conduit.
